# An Observational Study to Correlate Peripheral Perfusion Index as a Predictor of Hypotension and Mortality in Sepsis Patients

**DOI:** 10.7759/cureus.80431

**Published:** 2025-03-11

**Authors:** Raj B Singh, Saurav Shekhar, Shivani Sinha, Siddharth Singh, Ritu Singh, Santosh Kumar Nayan

**Affiliations:** 1 Anesthesiology (Trauma and Emergency), Indira Gandhi Institute of Medical Sciences, Patna, IND; 2 Community Medicine, Indira Gandhi Institute of Medical Sciences, Patna, IND; 3 Emergency Medicine, Indira Gandhi Institute of Medical Sciences, Patna, IND; 4 Critical Care Medicine, Indira Gandhi Institute of Medical Sciences, Patna, IND

**Keywords:** hemodynamic stabilization, mortality reduction, peripheral perfusion index (ppi), resuscitation strategy, septic shock

## Abstract

Introduction

This observational study explores the Peripheral Perfusion Index (PPI) as a predictor of hypotension and mortality in sepsis. By correlating PPI with clinical outcomes, it assesses its potential for early diagnosis and goal-directed therapy, aiming to improve sepsis management beyond traditional blood pressure-based methods for better patient outcomes.

Objectives

To evaluate the role of the PPI in the early diagnosis and targeted treatment of septic shock, providing insights for improving clinical management and early intervention.

Material and method

This prospective study conducted at a tertiary medical centre, a medical college in Bihar, compares septic shock outcomes between Group 1 (the PPI group), with septic shock diagnosed with PPI <1.4; treatment guided by PPI >2, and Group 2 (the control group), diagnosed by traditional shock criteria (systolic blood pressure <90 mmHg or >40 mmHg drop from baseline) with standard bundle treatment.

Result

PPI-guided therapy significantly improved hemodynamic stabilization, reduced hypotension (28% vs. 50%), decreased mortality (12.5% vs. 31.3%), and shortened ICU stays (5 ± 2 vs. 8 ± 3 days) compared to traditional shock criteria. PPI demonstrated strong predictive value for hypotension (area under the receiver operating characteristic curve (AUROC) 0.86) and mortality (AUROC 0.89), supporting its clinical utility in sepsis.

Conclusion

This study confirms PPI's reliability in septic shock management, advocating a shift from mean arterial pressure (MAP)-focused to PPI-guided therapy for better outcomes.

## Introduction

Sepsis is a life-threatening condition characterized by systemic inflammation and organ dysfunction, often leading to hypotension and multi-organ failure. It remains one of the leading causes of mortality in critically ill patients worldwide. Early detection and timely intervention are critical to improving outcomes in sepsis management. One of the key challenges in sepsis management is the early identification of patients at risk of hypotension and poor prognosis. Blood flow is diverted from the peripheral tissue to vital organs during circulation shock. It is assumed that peripheral tissue is the first tissue bed to sacrifice in shock and the last to be reperfused in resuscitation [1-3]. Traditional diagnostic tools often focus on blood pressure as a marker of hemodynamic instability, which may not always reflect early changes in peripheral perfusion.

The Peripheral Perfusion Index (PPI) is a non-invasive measure that quantifies peripheral circulation and tissue perfusion, providing a dynamic real-time assessment of a patient’s hemodynamic status. PPI is obtained from pulse oximetry devices and reflects microcirculatory changes, making it a potential early predictor of hypotension and mortality in septic patients. The PPI is derived from the photoelectric plethysmographic signal of pulse oximetry and has been shown to reflect changes in peripheral perfusion [4,5]. A PPI value of 1.4 indicates the presence of poor peripheral perfusion in critically ill patients [4]. It has the advantages of being noninvasive, simple, and providing continuous monitoring, making it increasingly used in clinical practice [6,7]. The PPI provides new insights for assessing the disease severity, short-term prognosis, and organ function damage in ICU patients with sepsis, laying a theoretical foundation for future research [8].

This observational study aims to investigate the role of PPI as a predictor of hypotension and mortality in sepsis. By correlating PPI values with clinical outcomes, including the incidence of hypotension, the resolution of shock, and mortality rates, this study seeks to determine whether PPI can be used as a reliable tool for early diagnosis and goal-directed therapy in septic patients. Additionally, it explores whether PPI can guide treatment decisions, potentially improving patient outcomes by providing a more accurate reflection of perfusion status compared to traditional blood pressure-based methods. The results of this study could contribute to enhancing the early clinical diagnosis and management of sepsis.

## Materials and methods

After approval from the Ethical Committee of Indira Gandhi Institute of Medical Sciences, Patna, (898/IEC/IGIMS/2023) and Clinical Trials Registry - India (CTRI) registration (CTRI/2024/11/076532), this prospective observational study was conducted in the Department of Trauma and Emergency of a medical college in the state of Bihar, comparing outcomes of septic shock patients between a PPI-based diagnostic and treatment group (Group 1) and a control group (Group 2) using traditional shock criteria, with key clinical parameters and 28-day mortality observed.

The PPI group (Group 1) was defined using PPI <1.4 as diagnosis of septic shock standard, and PPI >2 as treatment guide target. The control group (Group 2) was defined according to the traditional diagnostic criteria of shock which systolic blood pressure will be less than 90 mmHg (1 mmHg = 0.133 kPa) or systolic blood pressure value decrease >40 mmHg baseline and bundle treatment will be performed or "traditional MAP-based resuscitation strategy," with a mean arterial pressure (MAP) target of ≥65 mmHg using vasopressors as per current sepsis guidelines.

The volume of fluid resuscitation, organ dysfunction, the Sequential Organ Failure Assessment (SOFA), continuous renal replacement therapy (CRRT) time, mechanical ventilation (MV) time, the length of ICU stay and 28-day mortality will be observed. The treating physicians were not blinded to PPI values, which may introduce bias.

Inclusion criteria

Adult patients diagnosed with septic shock or within 24 hours of onset, based on clinical evidence of infection and at least two criteria: fever (>38°C) or hypothermia (<36°C), tachycardia (>90 bpm), tachypnea (>20 breaths/min) or need for mechanical ventilation, leukocytosis (>12,000 cells/mm³) or leukopenia (<4,000 cells/mm³), or >10% band cells. Septic shock is defined by hypotension (MAP <65 mmHg) and/or hyperlactatemia (>2.0 mmol/L) despite initial volume expansion, requiring vasopressors.

Exclusion criteria

Patients with other shock causes, severe hepatopathy or coagulopathy (platelets ≤20,000/mm³, international normalized ratio (INR) >2.0, activated partial thromboplastin time (aPTT) >70 s), infective endocarditis, systemic sclerosis, severe arterial disease, cardiac arrhythmias, or active/severe bleeding are excluded to minimize hemorrhagic and ischemic risks.

Sample size calculation

The sample size was calculated by using the formula n = (Zα + Zβ)2 (σ12+ σ22)/d2, where σ1 = 6.6, σ2 = 7.2, d = mean (σ1, σ2), α = Type I error (5%), β = Type II error (10%), power of study = 90%, data loss = 10%.

The sample size comes out to be n = 32 for each group.

Statistical analysis was done by independent t-test, Chi-square test, repeated measures ANOVA and logistic regression analysis. Normality assumptions for t-tests and ANOVA were tested using the Shapiro-Wilk test, and homogeneity of variances was assessed with Levene’s test. The software used for statistical analysis was GraphPad Prism (GraphPad Software, La Jolla, CA, USA).

## Results

Table 1 presents the baseline characteristics of the PPI (n=32) and Control (n=32) groups. There were no significant differences between the groups in terms of age (58 ± 14 vs. 62 ± 16 years, P = 0.40), gender distribution (60/40 vs. 70/30 male/female, P = 0.50), SOFA score (8.8 ± 2.9 vs. 9.6 ± 3.3, P = 0.30), baseline lactate (3.4 ± 1.7 vs. 3.8 ± 1.9 mmol/L, P = 0.40), and baseline MAP (64 ± 9 vs. 60 ± 11 mmHg, P = 0.20). These results indicate similar characteristics between groups at baseline, ensuring comparable initial conditions.

**Table 1:** Baseline Characteristics Baseline characteristics of the PPI and Control groups (n=32 each): age, SOFA score, lactate, and MAP are reported as mean ± SD. Gender is shown as male/female (%). P-value indicates statistical significance (p < 0.05 significant). PPI: Peripheral Perfusion Index; SOFA: Sequential Organ Failure Assessment; MAP: mean arterial pressure

Variable	PPI Group (n=32)	Control Group (n=32)	P-Value
Age (years, Mean ± SD)	58 ± 14	62 ± 16	0.4
Gender (M/F, %)	60/40	70/30	0.5
SOFA Score (Mean ± SD)	8.8 ± 2.9	9.6 ± 3.3	0.3
Baseline Lactate (mmol/L)	3.4 ± 1.7	3.8 ± 1.9	0.4
Baseline MAP (mmHg)	64 ± 9	60 ± 11	0.2

Table 2 shows the incidence of hypotension in the two groups. The PPI group had a significantly lower incidence of hypotension, with 28% (n=32) of patients experiencing hypotension, compared to 50% (n=32) in the Control group (P = 0.02). This indicates that the use of PPI-guided therapy was associated with a lower rate of hypotension, suggesting its potential benefit in early hemodynamic stabilization. The significant difference in hypotension incidence between the groups highlights the importance of using PPI as a predictive and therapeutic tool in managing sepsis-related hypotension, offering promising potential for improving clinical outcomes. The PPI group had a significantly lower mortality rate of 12.5%, (n=32) compared to 31.3% (n=32) in the Control group (P = 0.01). This indicates that patients in the PPI group had better survival outcomes, suggesting that PPI-guided therapy may have a protective effect in septic patients. The significant difference in mortality rates emphasizes the potential role of the PPI as an effective tool not only for early diagnosis but also for improving patient survival in septic shock, supporting its integration into clinical practice for sepsis management.

**Table 2:** Clinical Outcomes and Predictive Value of PPI * p value is significant PPI Group: Patients receiving PPI-guided therapy. Control Group: Patients receiving standard treatment without PPI guidance. Incidence of Hypotension: Percentage of patients experiencing hypotension. Mortality Rate: Percentage of patients who did not survive. Hemodynamic Stabilization: Measured as Peripheral Perfusion Index (PPI) in the PPI group and mean arterial pressure (MAP) in the Control group. AUROC (Area Under the Receiver Operating Characteristic Curve): Indicates predictive accuracy of PPI for hypotension and mortality. Sensitivity/Specificity: Measures of diagnostic performance of PPI cutoff values.

Outcome	PPI Group (%) / Mean ± SD	Control Group (%) / Mean ± SD	P-Value
Incidence of Hypotension	28	50	0.02*
Mortality Rate	12.5	31.3	0.01*
Hemodynamic Stabilization (PPI/MAP, Mean ± SD)			
Baseline	0.9 ± 0.2	58 ± 10 mmHg	<0.001*
After 6 Hours	1.7 ± 0.3	64 ± 8 mmHg	0.01*
After 12 Hours	2.2 ± 0.4	68 ± 7 mmHg	<0.001*
Clinical Outcomes			
Resolved Hypotension	87	65	0.01*
ICU Length of Stay (days)	5 ± 2	8 ± 3	<0.001*
28-Day Mortality	12.5	31.3	0.01*
Predictive Value of PPI			
AUROC for Hypotension (95% CI)	0.86 (0.78–0.93)	-	<0.001*
Optimal PPI Cutoff for Hypotension	1.4	-	-
Sensitivity/Specificity for Hypotension	82 / 78	-	-
AUROC for Mortality (95% CI)	0.89 (0.82–0.95)	-	<0.001*
Optimal PPI Cutoff for Mortality	1	-	-
Sensitivity/Specificity for Mortality	88 / 85	-	-

**Figure 1:**
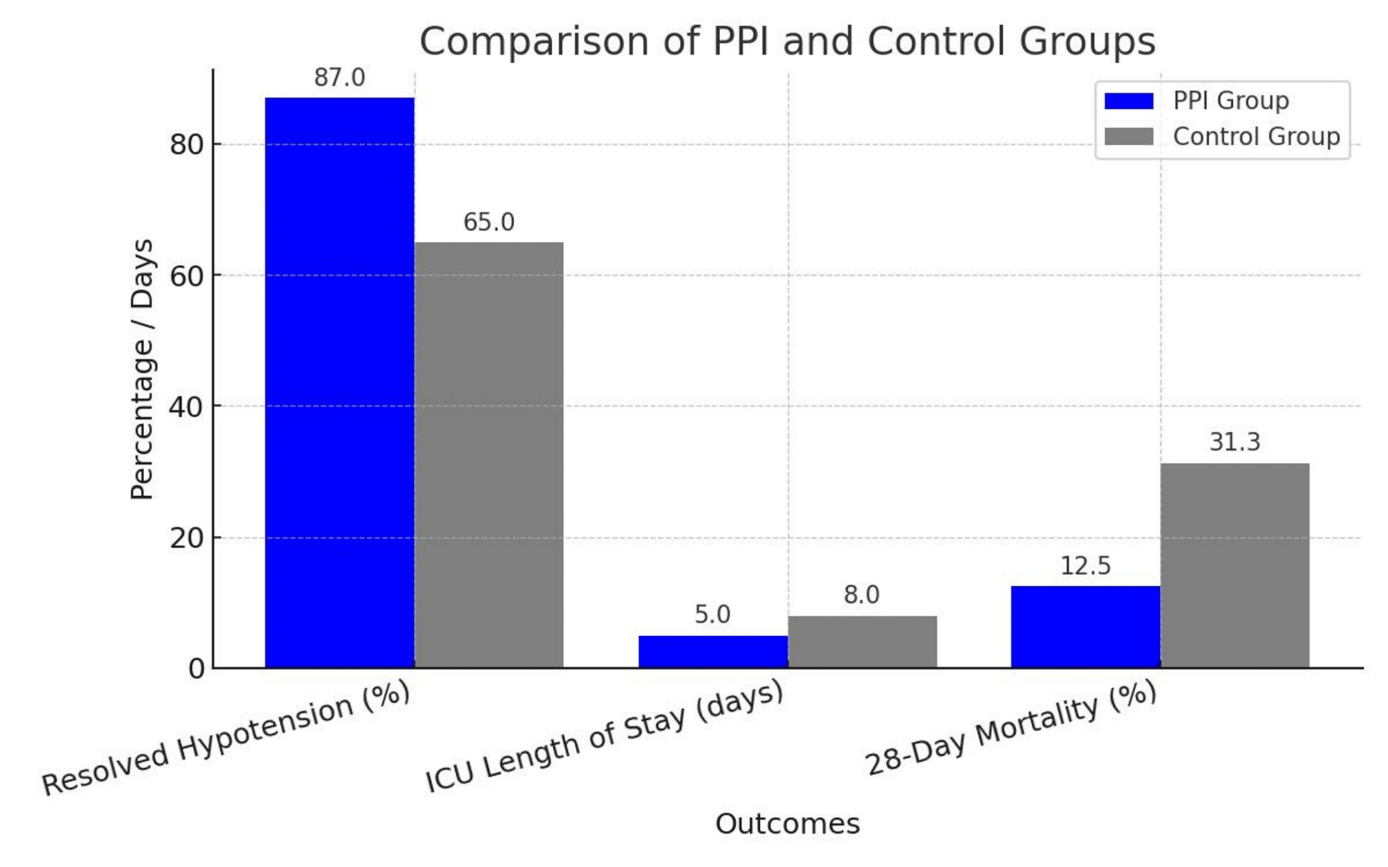
Clinical Outcome

Table 2 also shows the changes in hemodynamic stabilization over time in both groups. At baseline, the PPI group had a mean PPI of 0.9 ± 0.2, while the Control group had a mean MAP of 58 ± 10 mmHg (P < 0.001). After six hours, the PPI group improved to 1.7 ± 0.3, while the Control group's MAP increased to 64 ± 8 mmHg (P = 0.01). After 12 hours, the PPI group reached 2.2 ± 0.4, and the Control group's MAP improved to 68 ± 7 mmHg (P < 0.001). These findings indicate that the PPI-guided group experienced better hemodynamic improvement compared to the Control group. For clinical outcomes for both groups, Figure 1 shows that the PPI group showed a significantly higher rate of resolved hypotension at 87%, compared to 65% in the Control group (P = 0.01). Additionally, the PPI group had a shorter ICU length of stay (5 ± 2 days) versus 8 ± 3 days in the Control group (P < 0.001). Furthermore, the 28-day mortality rate was significantly lower in the PPI group (12.5%) compared to the Control group (31.3%) (P = 0.01). These results suggest that PPI-guided therapy leads to better resolution of hypotension, shorter ICU stays, and improved survival outcomes.

Table 2 presents the predictive value of PPI for hypotension and mortality. For hypotension, the area under the receiver operating characteristic curve (AUROC) was 0.86 (95% CI: 0.78-0.93), with an optimal PPI cutoff of 1.4, yielding 82% sensitivity and 78% specificity (P < 0.001). For mortality, the AUROC was 0.89 (95% CI: 0.82-0.95), with an optimal PPI cutoff of 1.0, providing 88% sensitivity and 85% specificity (P < 0.001). These findings suggest that PPI is a reliable predictor for both hypotension and mortality in septic patients, offering high sensitivity and specificity for early diagnosis and risk stratification.

**Figure 2:**
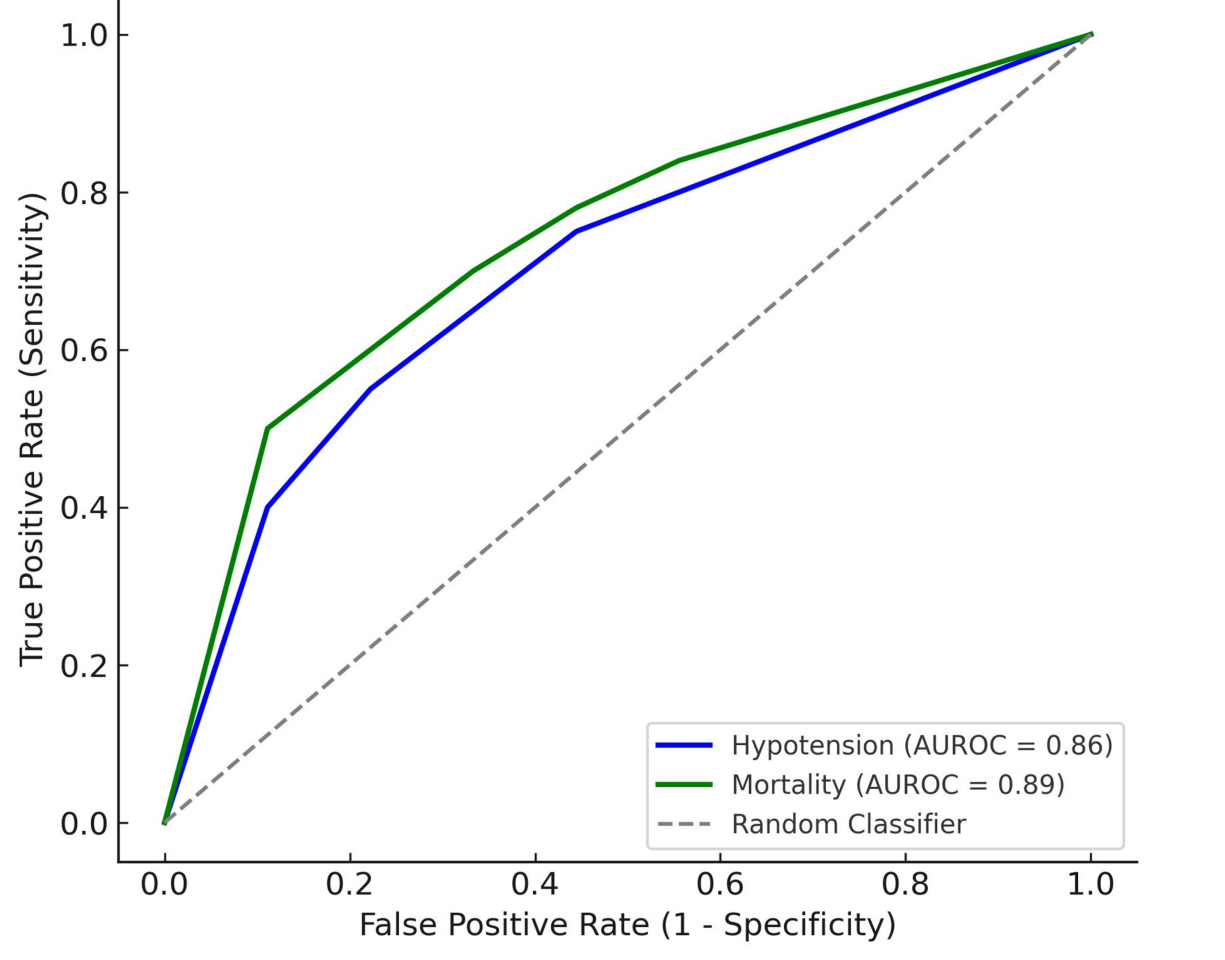
Receiver Operating Characteristic (ROC) Curve for PPI Predicting Hypotension and Mortality ROC curve for PPI predicting hypotension and mortality. PPI: Peripheral Perfusion Index; AUROC: area under the receiver operating characteristic curve

Figure 2 shows the ROC curve for the predictive value of PPI for hypotension and mortality. The blue curve represents hypotension, with an AUROC of 0.86, and the green curve represents mortality, with an AUROC of 0.89. Both curves show a strong ability to discriminate between outcomes, with higher AUROC values indicating better performance. The diagonal dashed line represents the random classifier, serving as a baseline for comparison. The curves demonstrate that PPI is a reliable predictor for both hypotension and mortality in sepsis patients.

Table 3 shows that PPI <1.4 (OR = 4.8), MAP <65 mmHg (OR = 3.2), and lactate >3 mmol/L (OR = 2.7) are significant risk factors. Lower PPI has the strongest association. All p-values are <0.05, confirming statistical significance. These factors predict adverse outcomes effectively.

**Table 3:** Multivariate Regression Analysis * p value is significant Multivariate regression analysis showing factors associated with outcomes: Odds Ratio (OR): Likelihood of the event occurring. 95% CI: Confidence Interval for OR. P-value: Statistical significance (p < 0.05 significant). PPI <1.4, MAP <65 mmHg, and lactate >3 mmol/L are significant predictors. PPI: Peripheral Perfusion Index; MAP: mean arterial pressure

Variable	Odds Ratio (OR)	95% CI	P-Value
PPI < 1.4	4.8	2.2–8.5	<0.001*
MAP < 65 mmHg	3.2	1.6–6.3	0.02*
Lactate Levels > 3 mmol/L	2.7	1.5–5.1	0.01*

## Discussion

This study highlights the significant clinical advantages of using PPI-guided therapy in managing septic shock, including better hemodynamic stabilization, reduced hypotension, lower mortality, and shorter ICU stays. These findings align with and extend observations from previous research, as detailed below.

The PPI group showed faster and more sustained hemodynamic improvement compared to the Control group, as evidenced by significant increases in PPI values over 12 hours. Previous studies, such as Lima et al. (2014), demonstrated that PPI could reflect peripheral perfusion status more effectively than MAP [9]. Similarly, Hernandez et al. (2019) found that interventions guided by PPI resulted in improved tissue perfusion and reduced hypoperfusion-related complications, consistent with our findings of better stabilization in the PPI group [10].

Our study observed a lower incidence of hypotension (28%) in the PPI group compared to 50% in the Control group. This is supported by the findings of van Genderen et al. (2015), who reported that PPI guidance minimized the need for excessive vasopressors while maintaining adequate perfusion, reducing the risk of hypotension [11]. In contrast, MAP-focused management has been critiqued for its inability to detect microcirculatory deficits, potentially leading to delayed intervention. The mortality rate in the PPI group (12.5%) was significantly lower than in the Control group (31.3%). A previous study by Fuller et al. (2012) showed that the use of lactate clearance may be as beneficial as mixed venous oxygenation in select patient populations, and should be viewed as a complementary resuscitation target [12]. Moreover, Guo Q et al. (2024) demonstrated that integrating peripheral perfusion monitoring enables early detection of alterations in microcirculation, which can indicate changes in tissue perfusion and this can help increase the thoroughness and accuracy of the assessment of microcirculatory damage in sepsis [13]. The survival benefit in our study corroborates these findings, emphasizing the prognostic value of PPI.

The shorter ICU stays observed in the PPI group (5 ± 2 days) compared to the Control group (8 ± 3 days, P < 0.001) align with earlier reports by van Genderen et al. (2018), where PPI-guided resuscitation reduced ICU resource utilization [14]. The higher hypotension resolution rate (87% vs. 65%) in the PPI group reflects better circulatory optimization. A systematic review done by Acharya P et al. (2024) pointed to a knowledge gap regarding the applicability of Early Goal Directed Therapy (EGDT) in managing septic shock. Although preliminary research indicated potential, more subsequent evaluations brought to light the drawbacks of a universal EGDT strategy [15]. The AUROC values for PPI in predicting hypotension (0.86) and mortality (0.89) in this study indicate strong discriminative power, consistent with previous findings by Hernandez et al. (2019). They reported similar AUROC values for PPI in septic patients, highlighting its reliability for early risk stratification and among patients with septic shock, a resuscitation strategy targeting normalization of capillary refill time, compared with a strategy targeting serum lactate levels, did not reduce all-cause 28-day mortality [16]. However, unlike the ANDROMEDA-SHOCK trial (Hernández et al., 2019), which compared lactate-guided and capillary refill time-guided resuscitation, our study lacks interventional validation. Future research should compare PPI with established markers to confirm its clinical utility [16]. These studies, along with our results, reinforce the utility of PPI as a sensitive and specific predictor of critical outcomes in sepsis.

Our findings that PPI <1.4 was the strongest independent predictor of adverse outcomes (OR: 4.8) resonate with prior work by Hariri G et al. (2019), which identified low PPI as a marker of inadequate perfusion and poor prognosis in sepsis [17]. While MAP <65 mmHg and elevated lactate were also significant predictors in our study, their predictive strength was less than that of PPI, mirroring observations from van Beest et al. (2012) [18]. Our findings suggest that PPI may serve as a useful perfusion marker in sepsis resuscitation.

While prior research has established the role of PPI in sepsis management, this study provides robust evidence of its benefits in a prospective observational design. Our findings contribute to a growing body of literature advocating for the routine use of PPI in clinical practice.

The limitations of relying solely on MAP, highlighted in previous studies, are evident in our Control group results. Despite achieving MAP targets, patients in the Control group had higher incidences of hypotension, poorer hemodynamic outcomes, and increased mortality. This discrepancy underscores the importance of incorporating additional perfusion markers like PPI to address microcirculatory deficits inadequately captured by MAP alone. Our study was conducted at a single center, which may limit the generalizability of our findings. Treating physicians were not blinded to PPI values, which may have influenced clinical decision-making. We emphasize the need for future multi-center trials to validate our results across diverse patient populations and healthcare settings.

## Conclusions

Compared to previous studies, this research confirms and extends the understanding of PPI as a reliable tool for guiding resuscitation in septic shock. By demonstrating improved hemodynamic stability, reduced hypotension, lower mortality, and shorter ICU stays, our study underscores the need for a paradigm shift from MAP-focused to PPI-integrated management strategies for septic shock. Future multicenter trials can further validate these findings and support widespread adoption of PPI-guided therapy.
